# Molecular docking analysis of juglone with parvulin-type PPiase PrsA from *Staphylococcus aureus*

**DOI:** 10.6026/97320630019048

**Published:** 2023-01-31

**Authors:** Laskar Dipten, Amenti, Mondal Rajkrishna

**Affiliations:** 1 Department of Biotechnology, Nagaland University, Dimapur, Nagaland-797112, India

**Keywords:** PPiase, Parvulin, juglone, *Staphylococcus aureus*, PrsA, competitive inhibitor

## Abstract

*Staphylococcus aureus* is an opportunistic pathogen that causes variety of infections range from mild skin diseases to life-threatening sepsis. It is also notorious for acquiring resistance to numerous antibiotics. Parvulin-type peptidyl-prolyl cis-trans isomerase (PPiase) domain containing PrsA protein is considered as an essential folding factor for secreted proteins of Gram-positive bacteria. Therefore, it is considered as a potential target for anti-staphylococcal drug discovery. Juglone, plant-derived 1,4-naphthoquinone, shows confirmed antitumor and antibacterial activities. Destruction of bacterial biofilm, inhibition of enzyme expression, degradation of nucleic acids, and other pathways are likely the major possible mechanisms for *Staphylococcus aureus* inactivation by juglone. Selective inhibition of parvulin type PPiase by juglone has been proven biochemically. However, detail structural information of parvulin-juglone interaction and mechanism of enzymatic inhibition till unexplored. Past hypothesis on inactivation of parvulin type PPiase due to covalent attachment of juglone molecules to its cysteine residues is not acceptable for the *S. aureus* PrsA parvulin domain as that lacks cysteine. Docking studies showed that juglone binds to the active site residues of *S. aureus* PrsA parvulin domain involved in enzymatic reaction. Active site conserved histidine residue of parvulin may be involved in juglone interaction as it was found to be the common interactive residue in majority of docking complexes. Data shows Juglone possibly inhibits parvulin type PPiase through competitive inhibition mechanism. Subtle differences of juglone interactions with other orthologous parvulin domains will help to develop semisynthetic drug with higher specificity against *S. aureus*.

## Background:

Multiple drug resistant *Staphylococcus aureus* strains are considered as global threat as they cause variety of diseases including life threatening sepsis [1,2]. *S. aureus* has great contribution to nosocomial and community-acquired infections [3]. In quest of anti-staphylococcal drug discovery, potential of naturally occurring naphthoquinone derivative juglone potential is increasingly apparent. Possible anti-staphylococcal mechanisms of juglone are destruction of bacterial biofilm, inhibition of enzyme expression, degradation of nucleic acids, and other pathways [4- 6]. Juglone also shows selective inhibition to the parvulin type peptidyl-prolyl cis-trans isomerase (PPiase) found both in bacteria and in eukaryotes. PPiase folds proteins by interconverting the cis and trans isomers of peptide bonds with the amino acid proline. Parvulin is the smallest known protein with prolyl isomerase activity compares to other two types of PPiase namely FKBP and cyclophilins [7]. In Gram-positive bacteria including *S. aureus* Parvulin-type peptidyl-prolyl cis-trans isomerase (PPIase) domain is present in PrsA protein along with flanking N- and C-terminal domains. PrsA protein is localized in periplasmic space and anchored to membrane lipid through its N-terminal cysteine residue [8,9]. PrsA is considered as a potential drug target because of its vital role in folding of proteins including enzymes important for formation of the cell wall and toxins [9]. ∼ 100 residues long parvulin domain has four-stranded antiparallel β-sheet core surrounded by four α-helices (βα3βαβ2 parvulin-fold) [10]. Parvulin domain of *S. aureus* PrsA shows structural similarity and juglone based inhibition with other parvulin type proteins from human (Pin1), *E. coli* and yeast (Ptf1/Ess1). Pin1, Ptf1/Ess1 and *E. coli* parvulin have conserved cysteine residue and inactivated parvulin contains cysteine covalently linked with juglone. Therefore, it is considered as a necessary condition for parvulin inactivation [11]. However, *S. aureus* PrsA parvulin domain does not harbour any cysteine residue and which is replaced by aspartate residue in the catalytic centre. This indicates that covalent attachment of juglone to cysteine residue is not sufficient condition for inactivation which is supported by phenomena of 5-fold faster modification of thiol group than the rate of enzyme inactivation in case of the reaction of *E. coli* parvulin and its Cys69Ala variant with juglone [11]. Although several structures of parvulin type proteins from different organisms were determined experimentally but none of the structures was available with juglone. To find out the structural and functional insight of parvulin-juglone interaction, we have done interaction studies of juglone with various structures of parvulin from different organisms through molecular docking.

## Material and Methods:

## Retrieval of sequence and structure of parvulin type PPiase proteins:

Sequence of PrsA protein (A6QI23) of *S. aureus* Newman strain, human Pin1(Q13526) and *E. coli* parvulin (P0A9L5) were retrieved from uniport database and presence of putative conserved domain was analyzed by Interpro tool [12] and the sequences were compared using multiple sequence alignment tool Clustal Omega [13]. Structures of *S. aureus* parvulin domain (PDB ID: 2JZV), human Pin1 (PDB ID: 1PIN) [14] and *E. coli* parvulin (PDB ID: 1JNS) [15] were downloaded from Protein Data Bank (PDB) and used for docking study.

## Prediction of *S. aureus* full length PrsA structure:

Three dimensional structures of *S. aureus* PrsA was predicted using RoseTTAFold tool [16]. All five RoseTTAFold resulted structures of PrsA were evaluated by structure validation parameters available in SAVES v6.0 (https://saves.mbi.ucla.edu/). Best structure as per the validation parameters was further refined energetically using GalaxyRefine web server [17] and designated as R_PrsA. PrsA structure (AF_PrsA) predicted by AlphaFold [18] also retrieved and compared with RoseTTAFold predicted structure after refinement.

## Molecular docking:

Juglone structure (CID: 3806) was retrieved from pubchem database. Docking experiments were performed using AutoDock Vina [19]. Centres at (3.243, 1.311, 0.928), (1.185, 5.152, -1.965), (-0.442 13.129 28.471), (25.047 7.930 41.844) and (-0.080 -0.267 0.417) were used for R_PrsA, AF_PrsA, 2JZV, 1PIN and 1JNS structures respectively with fixed Grid box (40 Å x 40 Å x 40 Å) in the docking experiments. Docking resulted structures were analyzed by PyMol and BIOVIA Discovery Studio Visualizer tools.

## Results and Discussion:

**Figure 1:**
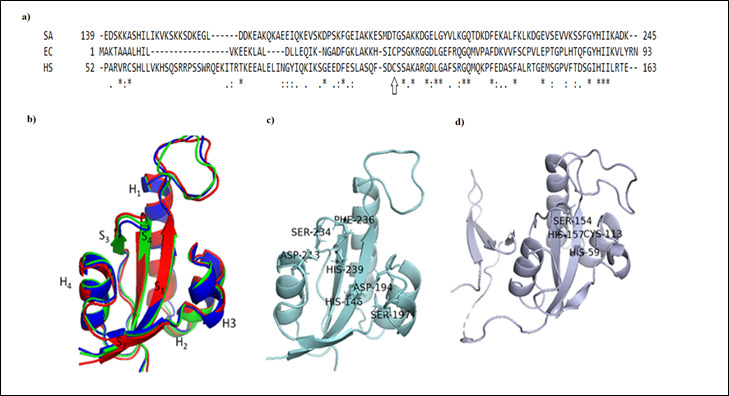
a) Multiple sequence alignment of parvulin domain sequences of *S aureus* PrsA(SA), human Pin1(HS) and *E. coli* Parvulin (EC), upward arrow showed the conserved cysteine residue in Pin1 and *E. coli* Parvulin but absent in PrsA. b) Superimposition of parvulin domain of R_PrsA(Blue), AF_PrsA (Green), 2JZV (RED) with conserved SH3SHS2 parvulin-fold c) and d) active site residues of *S. aureus* (PrsA) and human parvulin (Pin1) domain respectively.

**Figure 2:**
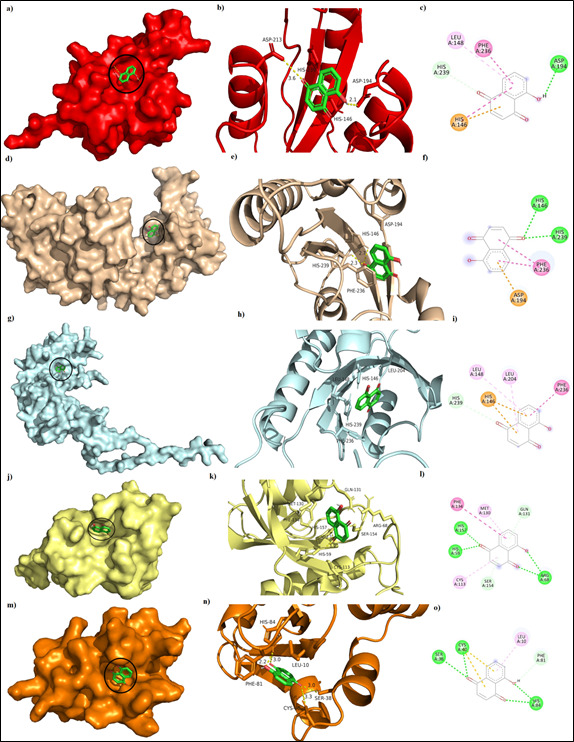
Interaction of juglone(green) with parvulin domains. a-c) 2JZV-juglone complex surface filling(a), ribbon structure representation(b) and key residues involved in interaction(c), d-f) R_PrsA -juglone complex surface filling(d), ribbon structure representation(e) and key residues involved in interaction(f), g-i) AF_PrsA -juglone complex surface filling(g), ribbon structure representation(h) and key residues involved in interaction(i), j-l) 1PIN-juglone complex surface filling(j), ribbon structure representation(k) and key residues involved in interaction(l), m-o) 1JNS-juglone complex surface filling(m), ribbon structure representation(n) and key residues involved in interaction(o).

The first objective of this study was to predict the three dimensional structure of full-length *S. aureus* PrsA protein using top two existing methods of protein structure prediction according to CASP14. Both the predicted structures were superimposed with structure of parvulin domain of PrsA determined by NMR spectroscopy to check the structural integrity of the parvulin domain in presence and absence of flanking N- and C-terminal domains. Secondly, all three structures were docked separately with ligand (juglone) to check the common pattern of inhibitor binding. Ligand binding study was extrapolated by considering parvulin from other organisms to come to a conclusion about the mechanism of action of parvulin inhibition by juglone and also to reveal structural insight of the interaction. Finally, to provide preliminary information that will help to design semi-synthetic juglone derivative drug specific for *S. aureus*. Sequence analysis of *S. aureus* PrsA protein by InterPro tool revealed presence of parvulin type domain (139-245) along with N and C-terminal flanking region similar like PrsA protein of Bacillus subtilis [7,8]. *S. aureus* parvulin domain showed 39.5% and 33.7% sequence similarity with *E. coli* parvulin and human Pin1 respectively and the conserved residues were highlighted in Figure 1a. Structure of full length *S. aureus* PrsA predicted by RoseTTAFold was further refined by Galaxyrefine tool and resulted structure was designated as R_PrsA. Superimposition of Parvulin domains from R_PrsA, AF_PrsA and 2JZV revealed the structural integrity of the domain structure in presence and absence of terminal domains as shown in Figure 1b. All of them retained typical parvulin domain structure comprised of four-stranded antiparallel β-sheet core surrounded by four α-helices (βα3βαβ2 parvulin-fold). Dissection of the active sites of *S. aureus* parvulin domain (2JZV) and human 1PIN showed that two histidine residues were conserved among both the proteins. However involvement of ASP194 was observed in case of 2JZV replacing the conserved active site cysteine residue of 1PIN (CYS113) and *E. coli* parvulin 1JNS (CYS40). Previous report based on MALDITOF-MS experiment claimed that the covalent attachment of juglone to cysteine residue was the necessary condition for parvulin inactivation [11]. However, absence of cysteine residue in *S. aureus* parvulin domain disproved the hypothesis. Therefore, to get a clue about parvuline-juglone interaction docking studies were performed considering juglone as ligand and different parvulin domain structures as receptor. Docking results showed common pattern for the involvement of HIS146, HIS239, ASP194, PHE236 and LEU148 residues of PrsA in the interaction with juglone. First four residues were considered as the key residues of active site for enzymatic reaction [10]. Docking studies were performed with juglone and 1PIN and IJNS separately to check whether juglone binding occurs to the active site residues of parvulin proteins from other organisms. Binding energy of the docked ligand at the active site of the 2JZV, R_PrsA, AF_PrsA, 1PIN and 1JNS were -5.0,-5.4,-5.2,-5.6 and -6.0 kcal/mol respectively. Active site key residues (HIS59, SER154, CYS113 and HIS157) of 1PIN had shown contribution to juglone binding as depicted in Figure 2. Interestingly juglone docking with 1JNS had shown (Figure 2m-o) the possible interaction of cysteine thiol group that revealed possibility of Michael addition of the thiol groups to juglone [11]. In brief, this study also proved that cysteine thiol group modification by covalent attachment of juglone may have very little contribution for inactivation of parvulin. However, involvement of active site residues of parvulin in juglone binding suggested juglone possibly compete with protein substrate (partially folded or misfolded) to bind enzymatic core of parvulin type PPiase. This study revealed the subtle differences observed in juglone binding with parvulin type PPiases from different organisms that can be extrapolated to design semisynthetic juglone derivative with specific antibacterial or antitumour potency.

## Conclusion:

Juglone is a plant derived 1,4-naphthoquinone with confirmed antitumor and antibacterial activities including *S. aureus*. It selectively inhibits *S. aureus* PrsA, a potential drug target, which plays vital role in folding of proteins including enzymes important for formation of the cell wall and toxins. Previous theory of inactivation of parvulin through permanent linkage of juglone to its cysteine residue is not universally applicable as parvulin domain of *S. aureus* and B. subtilis PrsA lack active site cysteine residue. This study showed the possible mechanism of parvulin (particularly for PrsA protein) inactivation by juglone as a competitive inhibitor. This work may give primary lead for the discovery of juglone derived semi-synthetic drug to control S. aureus infection including multiple drug resistant strains.
